# A new method using a vessel-sealing system provides coagulation effects to various types of bleeding with less thermal damage

**DOI:** 10.1007/s00464-020-08043-z

**Published:** 2020-10-15

**Authors:** Shosaburo Oyama, Takashi Nonaka, Keitaro Matsumoto, Daisuke Taniguchi, Yasumasa Hashimoto, Tomohiro Obata, Makoto Hisanaga, Masaaki Moriyama, Naoto Matsuo, Hideo Wada, Kiyoaki Hamada, Kouki Wakata, Tetsuro Tominaga, Shigekazu Hidaka, Terumitsu Sawai, Takeshi Nagayasu

**Affiliations:** 1 Department of Surgical Oncology, Nagasaki University Graduate School of Biomedical Sciences, 1-7-1 Sakamoto, Nagasaki, 852-8501 Japan; 2 Medical-Engineering Hybrid Professional Development Center, Nagasaki University Graduate School of Biomedical Sciences, Nagasaki, Japan

**Keywords:** Flat coagulation, Laparoscopic surgery, Thermal damage, Vessel-sealing system

## Abstract

**Background:**

Hemostasis is very important for a safe surgery, particularly in endoscopic surgery. Accordingly, in the last decade, vessel-sealing systems became popular as hemostatic devices. However, their use is limited due to thermal damage to organs, such as intestines and nerves. We developed a new method for safe coagulation using a vessel-sealing system, termed flat coagulation (FC). This study aimed to evaluate the efficacy of this new FC method compared to conventional coagulation methods.

**Methods:**

We evaluated the thermal damage caused by various energy devices, such as the vessel-sealing system (FC method using LigaSure™), ultrasonic scissors (Sonicision™), and monopolar electrosurgery (cut/coagulation/spray/soft coagulation (SC) mode), on porcine organs, including the small intestine and liver. Furthermore, we compared the hemostasis time between the FC method and conventional methods in the superficial bleeding model using porcine mesentery.

**Results:**

FC caused less thermal damage than monopolar electrosurgery’s SC mode in the porcine liver and small intestine (liver: mean depth of thermal damage, 1.91 ± 0.35 vs 3.37 ± 0.28 mm; *p* = 0.0015). In the superficial bleeding model, the hemostasis time of FC was significantly shorter than that of electrosurgery’s SC mode (mean, 19.54 ± 22.51 s vs 44.99 ± 21.18 s; *p* = 0.0046).

**Conclusion:**

This study showed that the FC method caused less thermal damage to porcine small intestine and liver than conventional methods. This FC method could provide easier and faster coagulation of superficial bleeds compared to that achieved by electrosurgery’s SC mode. Therefore, this study motivates for the use of this new method to achieve hemostasis with various types of bleeds involving internal organs during endoscopic surgeries.

**Electronic supplementary material:**

The online version of this article (10.1007/s00464-020-08043-z) contains supplementary material, which is available to authorized users.

The progress of endoscopic surgery in recent years has been remarkable. Among other reasons, this is due to the development of surgical equipment, particularly, hemostatic devices. During endoscopic surgery, hemostasis is vital because bleeding is not only the most common intraoperative complication, but also the most important determinant of the postoperative course [1]. Currently, several new hemostatic devices, such as vessel-sealing systems and ultrasonically activated scalpels, have been developed and have evolved the method used to achieve hemostasis during surgery. However, an appropriate hemostasis method for controlling superficial bleeds has not been identified. Conventional methods (e.g., monopolar and bipolar electrosurgery) are often challenging to obtain hemostasis safely and effectively during endoscopic surgery, especially for diffuse superficial bleeding, as this could lead to much collateral thermal spread.[2–8].

The LigaSure™ vessel-sealing device (Medtronic, Minneapolis, MN, USA) is often used during surgery because of its strong hemostatic power, low mist generated from the device, and low thermal effects [9]. In the conventional hemostatic method using LigaSure™, the bleeding site can be held and clamped between the tip blades (compression), and the bipolar function between the blades is activated [10]. However, certain surgical conditions make the use of LigaSure™ challenging. First, large, dense tissues cannot be sandwiched between the blades of the device. Second, clamp hemostasis for critical nerves and blood vessels with LigaSure™ can lead to nerve and blood vessel damage [11]. Thus, the use of this device is presumably difficult during standard procedures, such as cases of bleeding from the surface of big organs (e.g., pancreas, prostate, gallbladder, and neurovascular bundle), vascular surfaces, or perivascular areas.

To address these issues, we developed a new method of hemostasis using the vessel-sealing system LigaSure™, termed “flat coagulation” (FC). This technique is as follows: First, the vessel sealer is first open and activated. Then, the thermal energy generated on the backside of the blade is used for hemostasis. We found the FC especially useful for pelvic manipulation in rectal surgery.

Through clinical experiences by using this FC technique, we recognized a number of advantages if the LigaSure™ had been used at the beginning of the operation: (1) Immediate hemostasis was achieved at the bleeding site; (2) there was no need to replace the device, meaning that the surgical field was not cluttered with the cable of a new hemostatic device; and (3) there was no additional cost. On the other hand, this method requires an off-label use of the device. Therefore, the aim of this study was to evaluate the hemostatic efficacy of this FC method.

## Materials and methods

### Animals

Small, male mini-pigs, those that were Dai-Yorkshire, Landrace, and Duroc hybrid, were used (weight: approximately 30 kg) according to the animal experimental protocol approved by the local ethical and research institutional committee. Experimental animals were given ordinary feed. One day before the experiment, they were fasted and allowed to drink once. This study was carried out in strict accordance with the recommendations in the Guide for the Care and Use of Laboratory Animals of the National Institutes of Health. The protocol was approved by the Institutional Animal Care and Use Committee of Nagasaki University (Approval number 1607261327). All efforts were made to minimize suffering.

### Platform and settings of the desiccation and coagulation model in vivo

**Fig. 1:**
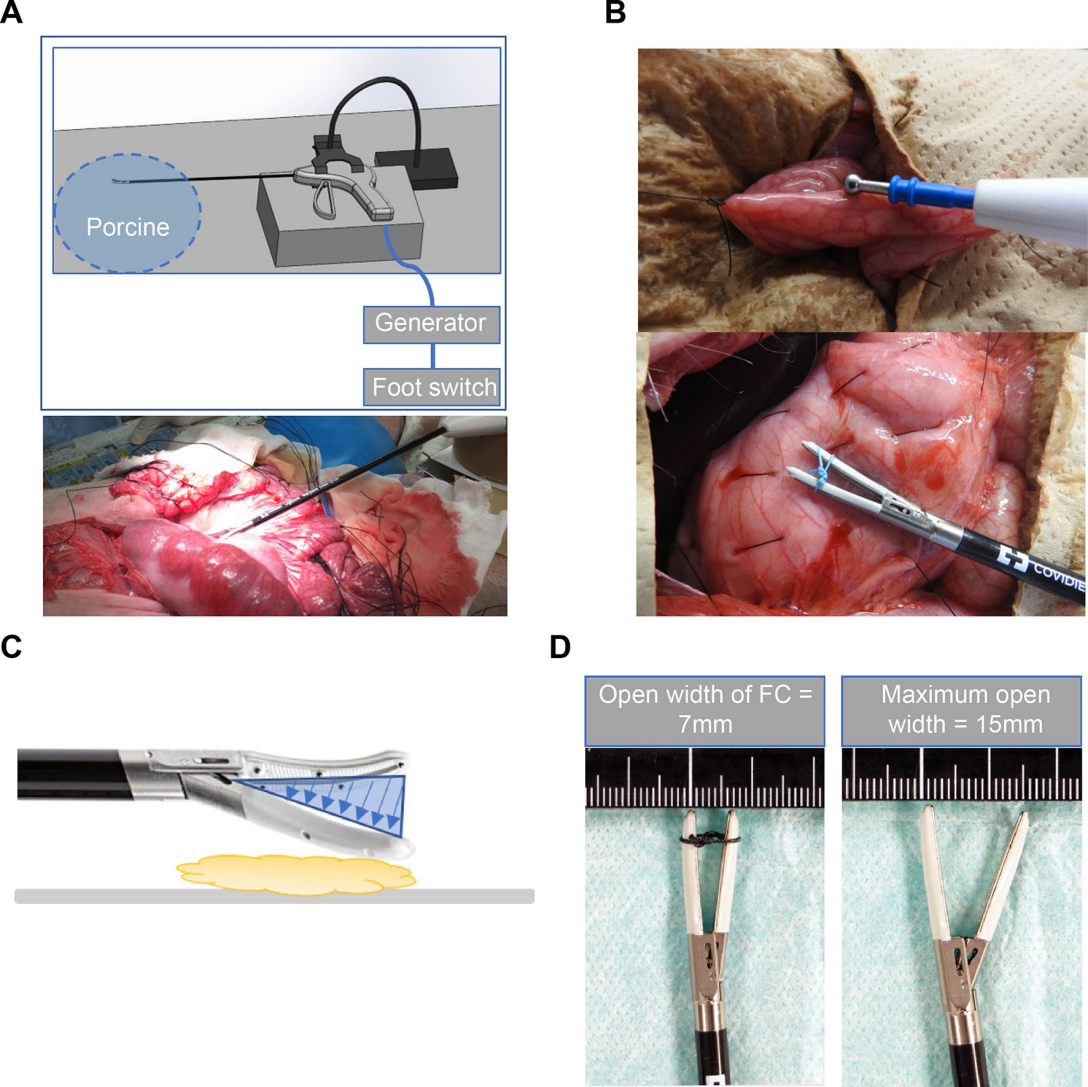
Experimental design. **A** Each device was fixed by the fixer’s arm on the stand. The generator used was ValleyLab FT10™ (MEDTRONIC). Desiccation of LigaSure™ flat coagulation (FC) was controlled by a foot switch. **B** Mechanism of FC. With the tip blade 7 mm open and the bipolar function is activated (blue triangle), the thermal energy can be made at the back (yellow cloud). **C** Actual experimental scenes (top, ball-type pole of electrosurgery of the soft coagulation mode; bottom, tip of the blade of LigaSure™ Maryland). **D** Width of LigaSure™’s blade: the narrow open width for FC is 7 mm, and the full open width is 15 mm

To examine the effects of energy devices, we conducted a comparative study using an electrosurgery, LigaSure™, and an ultrasonically activated scalpel. We used Valleylab™ FT10 energy platform (Medtronic, Minneapolis, MN, USA), which has the functions of both an electrosurgical scalpel and LigaSure™ platform. A cordless Sonicision™ (Medtronic, Minneapolis, MN, USA) was used as the ultrasonically activated scalpel. In the experimental setting, all devices were fixed, with the fixture’s arm having sufficient contact with the organ (Fig. 1A, B). Desiccation and coagulation was performed with no pressure applied to the tip. Electrosurgery and LigaSure™ were activated by the foot switch, while the ultrasonically activated scalpel was controlled by a hand switch. The coagulated tissue was dampened with 2 ml of normal saline at room temperature prior to the use of the FC using LigaSure™, electrosurgery’s soft coagulation (SC) mode, and Sonicision™.

### Monopolar electrosurgery

We chose and applied four types of electrosurgical modes as follows: cut mode, coagulation mode, spray mode, and SC mode. The actual clinical settings were as follows: the power settings in the cut, coagulation, and spray mode were set to 15 W, 25 W, and 35 W, and in the SC mode were set to 50 W, 60 W, and 70 W, respectively. The activation time in all modes was set to 1, 3, and 5 s. For the electrosurgical tip pole, the blade-type pole was used in the cut, coagulation, and spray mode and the ball-type pole was used in the SC mode.

### LigaSure™ FC

LigaSure™ FC is a method used for activating the bipolar function keeping the tip blades open and utilizing the thermal energy generated on the back of the blades to achieve hemostasis (Fig. 1C). The open width of the LigaSure™ blade was 7 mm, and the tip was fixed with a 2–0 silk thread (Fig. 1D). The maximum opening width of the blade was inappropriate for the experiment because air resistance between the electrodes was high, and the output was often erroneous. The application time settings were 1, 3, and 5 s, as with electrosurgery.

### Ultrasonically activated scalpel (Sonicision™)

We used the Sonicision™ ultrasonic scalpel in the low power mode. The performance time was set to 1, 3, and 5 s, just as before. Sonicision™ was adequately fixed, and the fixture’s arm had sufficient contact with the organ. The tip of the blades was closed and had fixed contact with the tissue, and it was controlled using the hand switch. In addition, the coagulation field was moistened with 2 ml of normal saline using an injection syringe.

### Operation

After anesthesia induction with ketamine (intramuscular, 20 mg/kg) and vecuronium (intravenous, 0.1 mg/kg), tracheotomy was performed, and maintenance anesthesia was administered by inhalation anesthesia with 2% isoflurane. After laparotomy with a mid-line abdominal incision, each device was tested in various organs and their effects examined. After the experiment, the organs were removed and animals were euthanized by pentobarbital overdose (150 mg/kg, div).

**Fig. 2:**
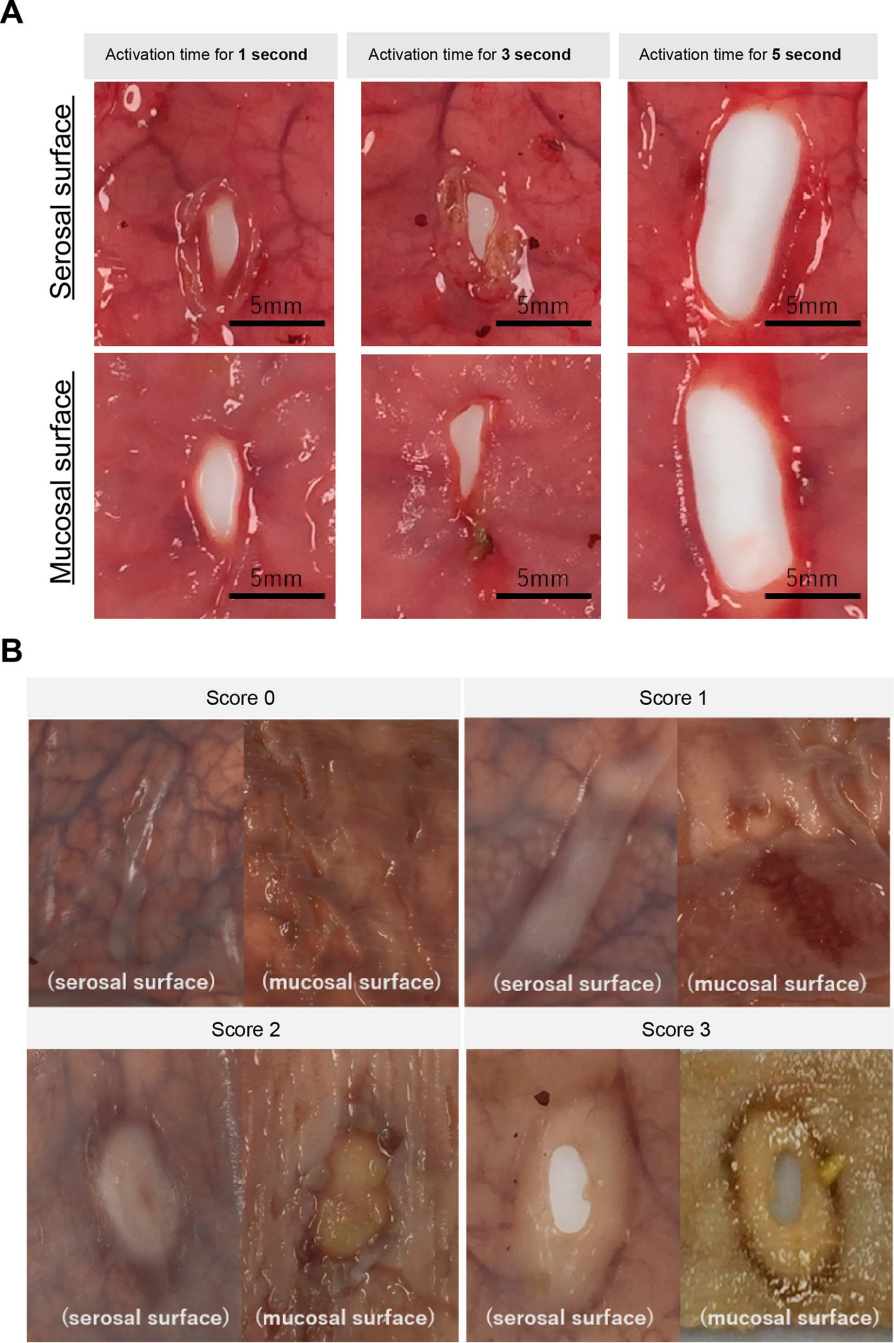
Desiccation and coagulation of porcine small intestine using devices. **A** Representative examples of macroscopic images of desiccated sites by electrosurgery (coagulation mode, 35 W). **B** A “damage score” of porcine small intestine by thermal damage of each energy device was developed (electrosurgery’s soft coagulation (SC) mode and LigaSure™ FC). We scored changes to the tissues on the opposite side of the desiccated site: 0, mucosal surface intact; 1, mucosal surface change ( +), not white; 2, mucosal surface change ( +), and white; 3, perforation

The small intestinal wall was marked and sectioned with a 10-cm interval between the sections (Fig. 1B). The middle wall section and the other side of the intestinal mesentery were coagulated using the devices. Desiccation and coagulation using each device were applied at each performance time and each power setting (*n* = 3). There were a total of 126 sections of the small intestines, and the degree of thermal injury was evaluated based on the mucosal surface changes on the contralateral side of the coagulated site (Fig. 2). From specimens that were coagulated in the small intestine, we chose the devices that did not perforate the tissues as hemostatic methods with little thermal effect. In the porcine small intestine, the following “damage score” that we created was used to select devices with less heat damage.

The liver was divided into 3 × 5 cm sections (total 48 sections), and the center of each section was coagulated with each device. Desiccation and coagulation were performed with each power and activation time (*n* = 4). The spread and depth of thermal damage were measured from these tissue specimens. Desiccation and coagulation using LigaSure™ FC was performed for 1 s, 3 s, and 5 s (*n* = 4). Desiccation and coagulation using electrosurgery’s SC mode was performed for 1 s, 3 s, and 5 s at 50 W, 60 W, and 70 W outputs, respectively (*n* = 4).

To examine the actual efficacy of LigaSure™ FC, a superficial bleeding porcine model in vivo was created, and the hemostasis time of LigaSure™ FC and electrosurgery’s SC was evaluated. The superficial bleeding porcine model was designed to have an oozing-like hemorrhage and was created by scratching one straight vessel near the small intestinal wall. We measured the time that the surgeons (*n* = 9) and medical students (*n* = 6) required to stop the bleeding when using LigaSure™ FC and electrosurgery’s SC mode (60 W), respectively. Measurements were performed three times, and average values were obtained. Immediately after the experiment, we evaluated the examinees using three questionnaires on a 5-point evaluation (1 point, bad; 5 points, good) based on the ease, effectiveness, and safety of the devices’ hemostatic procedures against the bleeding model.

### Morphological assessment using damage score

After fixing the cauterized intestine with formalin, we evaluated morphological changes of the serous and mucosal surfaces of the contralateral side of the small intestinal tract. We decided that the coagulated area on the serosa surface was unsuitable because there was a difference in the contact area due to the difference in the shape of the tip of each device. Regarding changes in the mucosal surface, we graded the degree of influence and damage to the small intestine: perforation was scored at 3; no perforation but the mucosal surface became white was scored at 2; no perforation but the mucosal surface became brown was scored at 1; and almost intact was scored at 0. Each device, power, and time zone were measured three times, and the scores were evaluated at a total of 126 locations.

### Histological assessment

The removed porcine organs were fixed with formalin, embedded in paraffin, sliced to 0.1 µm, and placed on a slide. Each slide was subjected to hematoxylin–eosin staining and Elastica van Gieson (EVG) staining. The slide specimen was blinded to the pathologist and evaluated histologically. The degree of thermal damage was evaluated by the depth of the tissue degeneration site due to heat produced at the cauterized site.

**Fig. 3:**
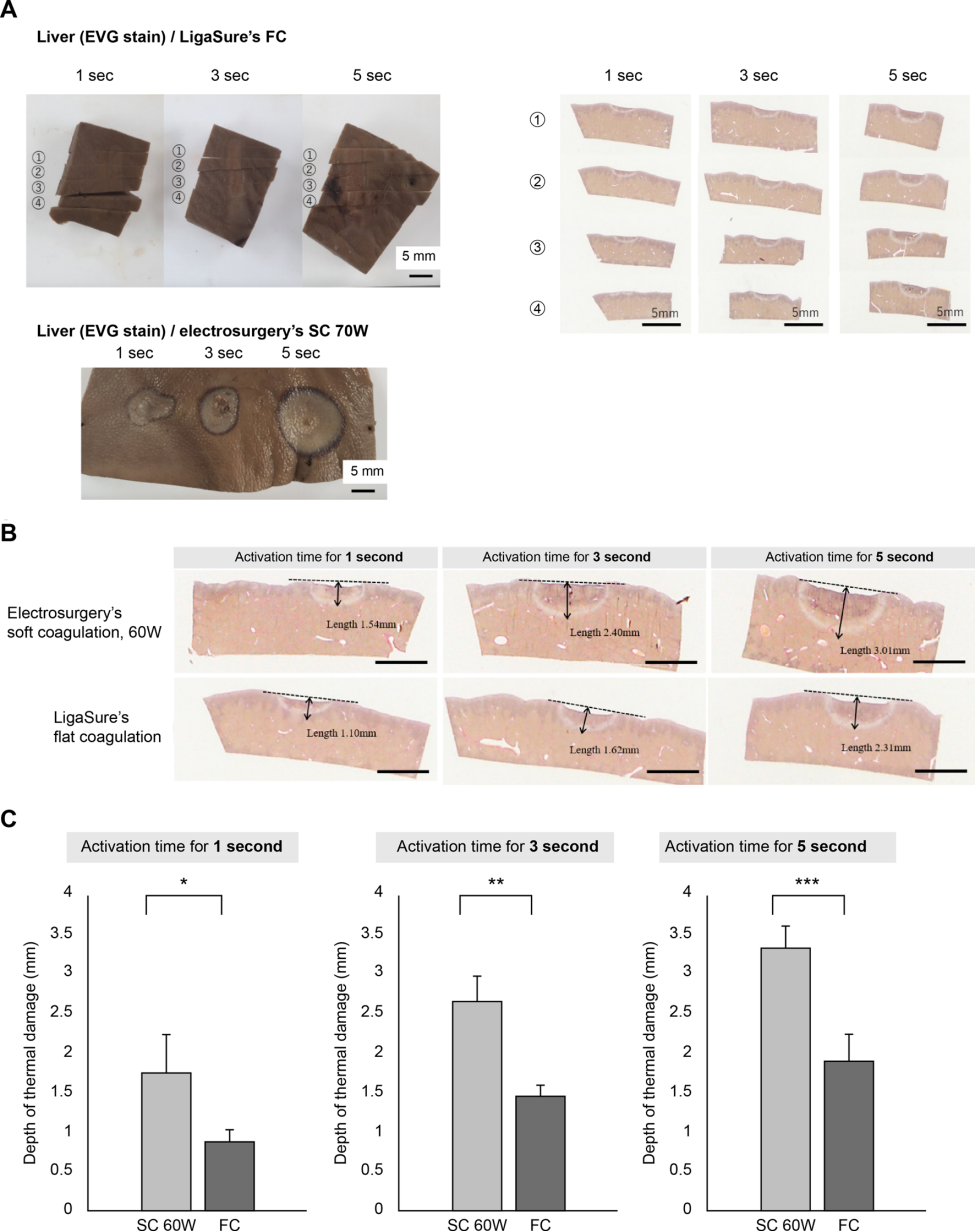
Assessment of flat coagulation in porcine stomach and liver. **A** The assessment of liver specimens of each device’s desiccation. Those of LigaSure™ FC were divided by 4 segment and examined (Left, up), and those of electrosurgery’s SC mode was evaluated on the maximum cleavage plane of the desiccated site (Left, bottom). The desiccation area of LigaSure™ FC had an elongated planar shape and uniform thermal damage was observed. **B** Representative histological specimens demonstrating thermal effect (scale bars, 3 mm). **C** Electrosurgery’s SC mode 60 W group (SC 60 W) has significantly more thermal damage than the LigaSure™’ FC group (FC) (*p* < 0.05)

We examined the depth and spread of the thermal damage in the liver because the porcine small intestine was too thin. After desiccation and coagulation with LigaSure™ FC, the coagulated part of the resected liver specimen was divided into 3-mm-wide sections along the minor axis (transverse to the major axis), and its cross section was evaluated histologically. The liver specimen desiccated and coagulated with electrosurgery’s SC mode was evaluated histologically on the maximum cleavage plane of the coagulated site (Fig. 3A). In addition, the depth of thermal damage in the liver was measured using Image J (NIH, USA). The liver specimens coagulated with LigaSure™ FC (total of 48 sites) and electrosurgery’s SC mode (total of 36 sites) were evaluated and assessed for differences in thermal damage between each burned site.

### Temperature measurements in vitro

Tissue surface temperature was measured with a super resolution infrared thermal imaging camera (InfReC R500, Nippon Avionics Co., Ltd) at the point of the device and around the device. We recorded a 5-s movie using this camera during desiccation and coagulation of a fresh pig’s meat block using LigaSure™ FC, electrosurgery’s SC mode (70 W), and Sonicision™ (low power mode). We performed each method three times and the average value was calculated from recorded data such as the maximum temperature of the coagulated parts, time to reach the maximum temperature, and point of the maximum temperature.

### Statistical assessment

All values are expressed as mean ± SD. Statistical analyses were performed using Student’s *t*-test. *P*-values < 0.05 were considered statistically significant. Data were analyzed using the software of an advanced online analysis program, InfReC Analyzer NS9500 Professional (Nippon Avionics Co., Ltd, Tokyo, Japan).

## Results

### Thermal damage by each energy device (device selection for the comparison group)

The porcine small intestinal wall thickness was less than 2 mm. In the cut/coagulation/spray mode of electrosurgery, the small intestine was obviously perforated at the 1-s desiccation and coagulation. However, in all modes in the LigaSure™ FC and most electrosurgery’s SC mode groups, there was clearly no histological perforation, although the thermal effects were observed up to the mucosal surface. In addition, the longer desiccation and coagulation time and higher output power in any device resulted in greater thermal effects on the porcine intestine (Fig. 2A and Table 1). Moreover, the in vivo experimental conditions could not be replicated for Sonicision™; thus, results could not be determined for this device [12]. Therefore, the LigaSure™ FC was compared with the electrosurgery’s SC mode.

**Table 1:**
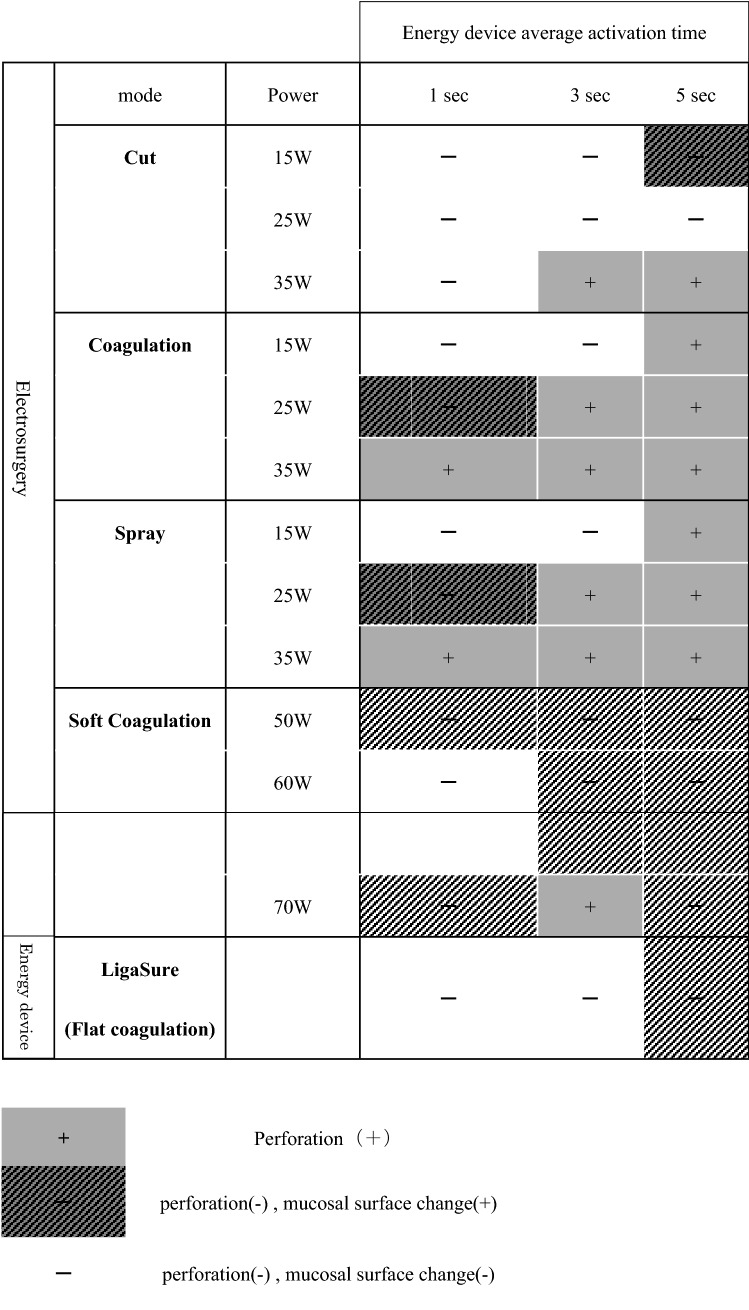
Desiccation and coagulation of porcine small intestines using devices

The degrees of thermal damage by each device. LigaSure™ flat coagulation (FC) had less of an effect on the small intestine

### Comparison of thermal damage in the small intestine

In LigaSure™ FC and almost all electrosurgery’s SC modes, no perforation was observed in any activation time, except in a single case in which electrosurgery’s SC mode was used at 60 W. FC had a damage score of 0 at 1 s, and the score gradually increased after 3 s. Although these scores tended to be lower than the damage scores obtained with the electrosurgery SC mode, no significant difference was found (damage score at 3 s, LigaSure™ FC vs electrosurgery’s SC mode 60 W, 1.33 vs 2.0; *p* = 0.184) (Fig. 2B and Table 2). The porcine small intestinal wall was too thin, and it was difficult to evaluate histologically.

**Table 2:** Damage score shows that LigaSure™ FC has less of an effect than electrosurgery’s SC mode

Device	Power	Energy device average activation time		
		1 s (*n* = 3)	3 s (*n* = 3)	5 s (*n* = 3)
	50 W	2	2	2
Electrosurgery (Soft coagulation)	60 W	1.33 ± 0.47	2	2
	70 W	2	2.33 ± 0.47	2
LigaSure (Flat coagulation)		0	1.33 ± 0.47	1.67 ± 0.47

LigaSure™ FC has a gradually increasing damage score after 3 s, but it is lower than that of electrosurgery’s SC mode (damage score at 3 s, LigaSure™ FC vs electrosurgery’s SC mode 60 W, 1.33 vs 2.0; *p* = 0.184)

### Expansion of the thermal damage in the liver

The depth of thermal damage in the liver showed that electrosurgery’s SC mode caused significantly more thermal damage than LigaSure™ FC (Fig. 3). In FC, the thermal impact appeared slightly stronger at the root of the LigaSure™ blade than at the blade tip, but the depth of thermal damage was almost the same, < 1 mm in 1 s, < 2 mm in 3 s, and about 2 mm in 5 s (Fig. 3A). In electrosurgery’s SC mode, a power of 50 W created deeper thermal damage than 60 W and 70 W, and the average depth of thermal damage was 3.34 ± 0.28 mm at 50 W for 5 s, 3.37 ± 0.28 mm at 60 W for 5 s, and 3.12 ± 0.98 mm at 70 W for 5 s. In addition, no statistically significant difference in the degree of thermal damage was observed between the electrosurgery’s SC mode at 60 W for 1 s (1.76 ± 0.47 mm) and LigaSure™ FC for 5 s (1.91 ± 0.35 mm) (*p* = 0.685) (Fig. 3B, C).

### Hemostasis of the superficial bleeding in porcine

**Fig. 4:**
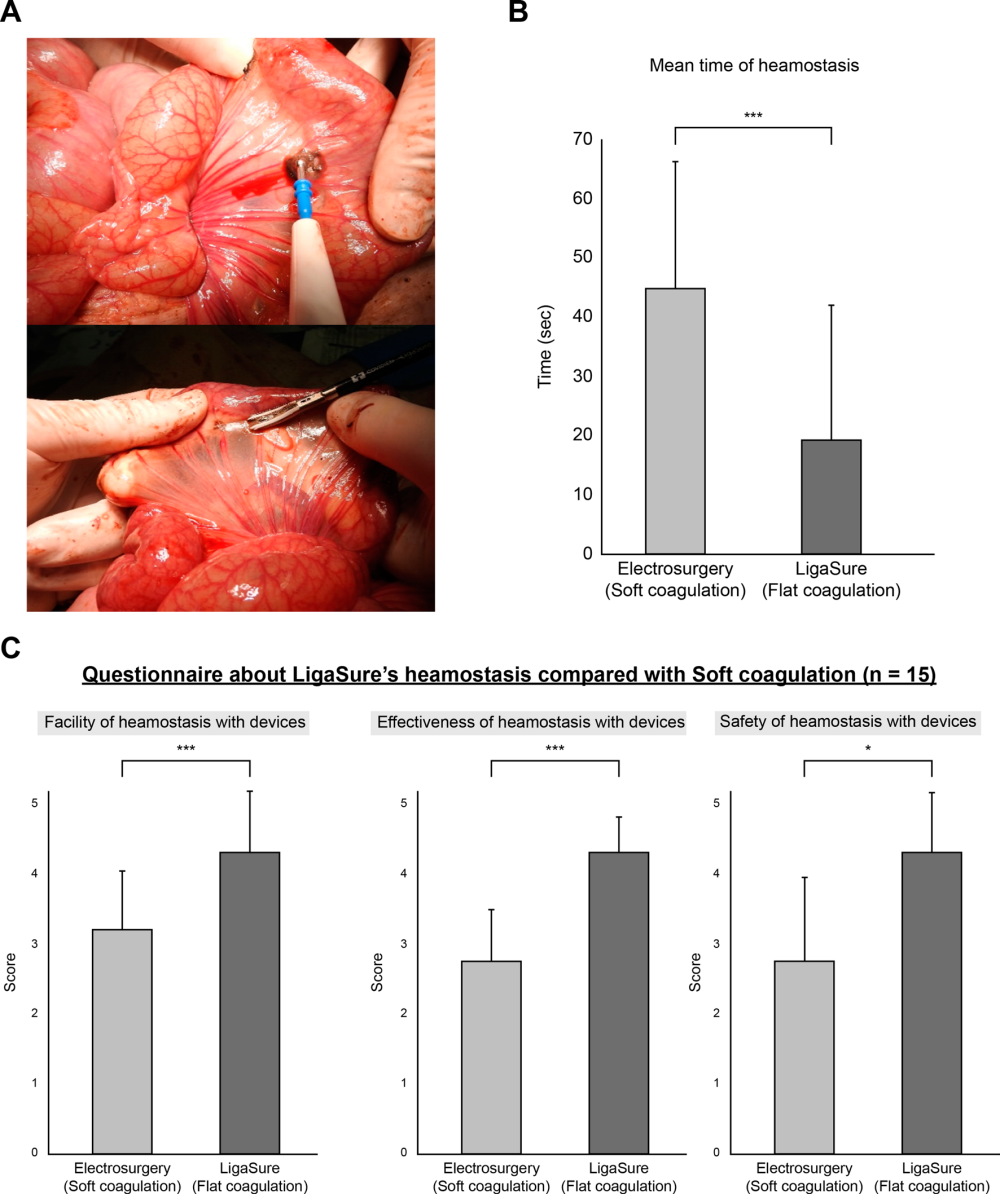
Superficial bleeding model in porcine mesentery. **A** Actual scenes of hemostatic methods in porcine superficial bleeding model (top, scene of electrosurgery’s SC mode; bottom, LigaSure™ FC). **B** The mean bleeding time in LigaSure™ FC was 19.54 ± 23.30 s and that in electrosurgery’s SC mode was 42.90 ± 21.92 s (*p* = 0.0046), and **C** even in the questionnaire, the effectiveness and safety were higher in LigaSure™ FC for the manipulation in the bleeding model

Both LigaSure™ FC and electrosurgery’s SC mode could stop bleeding completely (Fig. 4A). The mean hemostatic time in the FC group was 19.54 ± 22.51 s, and the mean time in the electrosurgery’s SC mode group was 44.99 ± 21.18 s (Fig. 4B) (Supplementary video 1). The hemostatic time was shorter in FC than in electrosurgery’s SC mode (*p* = 0.0046). Moreover, the hemostatic time achieved by the medical students using FC was shorter than that of the surgeon’s using electrosurgery’s SC mode, although the surgeons achieved a shorter hemostatic time than the medical students (mean hemostatic time: in LigaSure™ FC, surgeons vs medical students, 10.46 ± 4.61 vs 32.09 ± 31.14 s; in electrosurgery’s SC mode, surgeons vs medical students, 35.95 ± 16.16 vs 53.74 ± 21.75 s). In an experiment questionnaire comparing hemostasis in the superficial bleeding model, the usability, effectiveness, and safety were higher in the FC group (Fig. 4C).

### Thermography of each device using porcine in vitro model

**Fig. 5:**
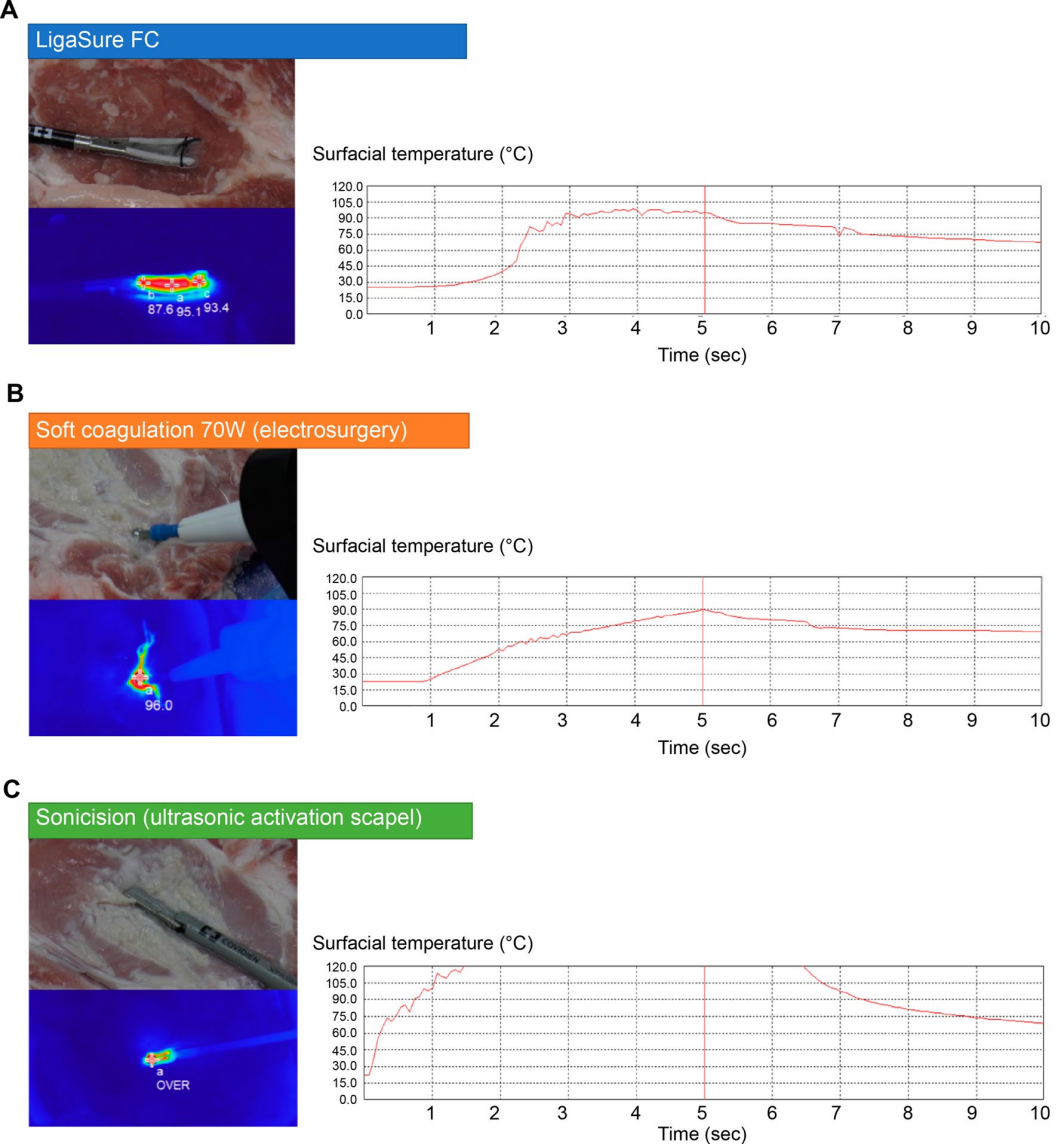
Thermography of superficial and surrounding temperatures using each device. The activation time was 5 s. **A** In the LigaSure™ FC, the temperature increased to around 90 °C quickly at about 2 s, and was maintained at less than 100 °C. **B** In electrosurgery’s SC mode, the surface temperature increased gradually, and the maximum temperature was 98.23 ± 14.88 °C at 5 s. **C** In Sonicision™, the temperature increased to more than 120 °C quickly in about 2 s

We examined the change in superficial temperature around each device during the application time of 5 s using a fresh pig’s meat block. In LigaSure™ FC, the temperature increased to around 90 °C quickly at about 2 s and was maintained at less than 100 °C (Fig. 5A). The maximum temperature with FC was 98.62 ± 0.55 °C at 5 s. The time taken to reach a stable temperature was 1.84 ± 0.48 s. In electrosurgery’s SC mode, the surface temperature increased gradually, and the maximum temperature was 96.63 ± 11.52 °C at 5 s (Fig. 5B). In Sonicision™, the temperature reached more than 120 °C quickly in about 2 s and was maintained above 120 °C for 2.76 ± 1.64 s (Fig. 5C).

## Discussion

Our study showed that a new hemostatic method, Flat coagulation method using LigaSure™, was effective for hemostasis of superficial bleeding in various organs, which is often observed in endoscopic surgeries. FC method using vessel-seal system was found to cause less of an effect on organs than conventional hemostasis methods.

We found two advantages of FC method. First, FC caused less thermal damage to organs compared to conventional coagulation methods. As shown in the liver desiccation and coagulation model, the coagulated area had an elongated planar shape, and uniform thermal damage was observed with FC. Although the depth of thermal damage was greater over time with FC, it produced less overall thermal damage than the electrosurgery’s SC mode. FC has lateral thermal effects comparable to conventional LigaSure™. Some reports supported our data by stating that the extent of lateral thermal damage by conventional LigaSure™ was approximately 1.5 mm [5–7]. In the thermographic results, the surface temperature of FC was not much different from that of electrosurgery’s SC mode. However, as can be seen from the lateral thermal effect to the liver, FC should be used for hemostasis only on the surface by creating more tangential energy flow to the hemostasis surface.

Second, FC achieves hemostasis of superficial bleeds faster and easier than electrosurgery’s SC mode. FC could create an environment in which it is easier to stop bleeding by activating the bipolar function just above and around the bleeding point. In the thermography, FC could generate a temperature environment of 90 °C to < 100 °C quickly around the bleeding point in about 2 s. LigaSure™ 's sealing system did not produce excessive heat due to the controlled power output of the generator (such as FT 10). This finding suggested that FC could be effective for planar hemostasis. In fact, our data showed that the hemostatic time by FC was much shorter than that of electrosurgery’s SC mode. The average hemostatic time of medical students using FC was less than that by surgeons using electrosurgery’s SC mode. Thus, regarding hemostasis using FC, it appeared that there was not much difference between mature and immature surgeons. In addition, the results of the questionnaire about hemostasis for the superficial bleeding supported that maneuverability and effectiveness of FC could be higher than that of electrosurgery’s SC mode.

The device does not only control point bleeds, but also superficial oozing [11, 13–18], which may be more challenging to do as a longer time of coagulation is often required to attain hemostasis. We used FC in the clinical setting of colorectal surgery. We realized that it is highly effective for hemostasis of superficial bleeding around blood vessels and neurovascular bundles, particularly during total pelvic exenterations and lateral lymphadenectomies in pelvic surgery (Supplementary video 2). Conventional hemostasis with electrosurgery generates “point” hemostasis at the tip of the electrosurgery, whereas this new FC method provides broad “planar” hemostasis. Furthermore, the manipulation of FC is easy due to its activation by simply opening the blade and desiccating and coagulating using a sweeping motion. This also helps to reduce the number of devices required during surgery.

There are several limitations in this study. First, FC may not be an appropriate hemostatic method in some cases, especially liver or renal injury [13–15]. In these cases, the electrosurgery’s SC mode could be more appropriate than FC because bleeding from the parenchymatous organ has a high risk of rebleeding [16]. However, there are some reports about thermal damage and complications due to thermal energy by electrosurgery’s SC mode; therefore, the operator should pay careful attention to this when using the device [19–24]. The second limitation is that our data were obtained from porcine organs, not human organs. Third, this study does not allow hemostasis by FC on the intestinal wall. Fourth, the damage score in this study was only used for scoring the macroscopic evaluation; therefore, the difference in the scores does not correlate with clinical results (e.g., perforation). Finally, LigaSure™ itself is an expensive device. Although FC is a very useful method during laparoscopic surgery with LigaSure™, an intraoperative hemostatic method must be selected in consideration of the cost-effectiveness. In the future, we believe that all surgeons can safely perform difficult surgery by utilizing this FC technology.

In conclusion, bleeding in organs can be effectively controlled with FC. Surgeons should select the appropriate hemostatic device for each case. FC can be a suitable hemostatic device in endoscopic surgery. This study demonstrates the potential of bipolar function rather than the characteristics of this vessel sealer. In doing so, this study helps expand our understanding of the LigeSure™ device and contributes to the potential development of endoscopic surgery.

## Electronic supplementary material

Below is the link to the electronic supplementary material.

Supplementary file1 Supplementary Video 1 Superficial bleeding model (MP4 101831 kb)

Supplementary file2 Supplementary Video 2 Operation video using LigaSureTM flat coagulation (WMV 150429 kb)
